# Full and Partial Facial Affect Recognition in Pediatric Brain Tumour Survivors and Typically Developing Children Following COVID-19 Pandemic

**DOI:** 10.3390/curroncol31080339

**Published:** 2024-08-09

**Authors:** Laurianne Buron, Sébastien Perreault, Serge Sultan, Marco Bonanno, Hallie Coltin, Caroline Laverdière, Émélie Rondeau, Leandra Desjardins

**Affiliations:** 1 Department of Psychology, Université de Montréal, 2900 Bd Édouard-Montpetit, Montreal, QC H3T 1J4, Canada; 2 Sainte-Justine’s University Health Center, 3175 Chem. de la Côte-Sainte-Catherine, Montreal, QC H3T 1C5, Canada; 3 Department of Neurosciences, Université de Montréal, 2900 Bd Édouard-Montpetit, Montreal, QC H3T 1J4, Canada; 4 Department of Pediatrics, Université de Montréal, 2900 Bd Édouard-Montpetit, Montreal, QC H3T 1J4, Canada

**Keywords:** social competence, facial affect recognition, pediatric brain tumour, survivorship

## Abstract

Affect recognition has emerged as a potential mechanism underlying the social competence challenges experienced by pediatric brain tumour survivors (PBTSs). However, many social interactions were altered during the pandemic, with the widespread use of masking potentially impacting affect recognition abilities. Here, we examine affect recognition in PBTSs and typically developing youth (TD) after the onset of the global pandemic. Twenty-three PBTSs and 24 TD between 8 and 16 years old were recruited and completed two performance-based affect recognition tasks (full and partial facial features) and a self-reported questionnaire on mask exposure in their social interactions. Their parents completed parent proxy questionnaires on their child’s social adjustment and sociodemographics. The scores between the PBTSs and TD did not differ significantly in full (*t*(45) = 1.33, *p* = 0.19, *d* = 0.39, 95% CI [−0.69, 3.40]) or partial (*t*(37.36) = 1.56, *p* = 0.13, *d* = 0.46, 95% CI [−0.47, 3.60]) affect recognition, suggesting similar affect recognition between the two groups. These skills were also not significantly correlated with social adjustment or mask exposure (*p* > 0.05). However, the combined sample had significantly better scores in affect recognition when exposed to partial facial cues versus full. Additionally, participants obtained lower scores on a measure of full facial affect recognition and higher scores on a measure of partial affect recognition compared to pre-pandemic data. The pandemic may have influenced affect recognition across youth, underscoring the importance of further research into its lasting impact on the social competence of youth.

## 1. Introduction

Progress in diagnosis and medical treatments has led to a growing population of pediatric brain tumour survivors (PBTSs) and a need to focus on the quality of their survivorship. Unfortunately, PBTS survivorship may be impacted by a broad range of disease and treatment late effects [1], with social competence emerging as a significant area of impairment, e.g., [2,3]. Notably, caregivers of PBTSs have reported social difficulties as the most devastating consequence of their child’s diagnosis [4]. PBTSs are also often described by caregivers, peers, and teachers as socially isolated, e.g., [5,6]. Risk factors for greater deficits in social competence in PBTSs include cranial radiation therapy, younger age at diagnosis, and longer time since diagnosis, e.g., [2,3].

Yeates et al.’s [7] social competence model, initially developed for children with brain disorders, has been directly applied as a framework to better understand PBTS’ social competence [2]. The model posits that social competence is a construct encompassing three main components: social information processing (SIP, i.e., the interpretation of social cues), social interactions (i.e., behaviours carried out in social situations), and social adjustment (i.e., perception of age-appropriate social competence) [7]. It is well established that PBTSs often experience difficulties in social adjustment, e.g., [2,3]. SIP may be an especially relevant component to attend to as it is foundational to the model and subsequently influences social interaction and social adjustment [7].

One specific element of SIP, facial affect recognition, has emerged in recent years as a key component toward better understanding social competence in PBTSs [2]. Facial affect recognition is the ability to properly recognize, discriminate, and interpret different affects (e.g., happy, sad, angry) demonstrated by someone’s facial expression. Correctly interpreting facial expressions, one of the richest sources of social nonverbal information [8,9], is essential for people to understand the social situations they are in and how to respond appropriately [9]. 

There is a growing number of studies examining facial affect recognition in PBTSs of different ages and times since diagnosis and using a variety of measures, e.g., [10,11]. PBTSs have been found to make more errors in recognizing adult facial expressions compared to children with Juvenile Rheumatoid Arthritis [10] and to obtain lower scores of affect recognition relative to typically developing children, TD [12]. Worse affect recognition was also found to be correlated with lower PBTS’ social adjustment as rated by parents [10] and with greater difficulty in naming a friend [13]. Hocking et al. [11] noted that PBTSs with worse levels of social adjustment made more errors in identifying facial affect compared to both TD and youth with autism spectrum disorder (ASD), concluding that facial affect recognition might be uniquely important to PBTS’ social competence compared to other children. On the other hand, two recent studies found that PBTSs did not differ in affect recognition compared to TD [11] and children with non-central nervous system solid tumours [14]. Discrepancies in findings may be explained by the variability in measures, tasks, and approaches used across studies, but they also highlight the need for the further study of affect recognition in PBTSs.

In addition to discrepancies in findings, there is currently a lack of studies considering the possible impact of the current COVID-19 pandemic and widespread mask use on affect recognition in PBTSs. With mask-wearing, only the upper portion of facial social cues (i.e., eyes, eyebrows, and forehead) is visible to infer emotions during social interactions. Thus, mask-wearing (less exposure to facial cues) may negatively influence affect recognition. Very few existing measures are available to address this inquiry, with one being the Reading the Mind in the Eye Test (RMET). The RMET is a measure evaluating a person’s ability to determine others’ emotions based solely on static images, without context, of the eye and upper eye region. Although it is frequently used as a measure of theory of mind (inferring mental states of others), the RMET demonstrates the quality of a facial affect recognition task [15,16]. The RMET has been previously used as a measure of emotion recognition, e.g., [17], and utilized in a study with children with brain injury [18], suggesting it could be administered to PBTSs for affect recognition purposes.

Overall, there is evidence that affect recognition is a relevant construct used to understand social competence in PBTSs, e.g., [10,12]. However, there are still many unknowns about (1) full and partial affect recognition in PBTSs compared to TD, (2) how it may relate to social adjustment, and (3) affect recognition in PBTSs in the context of the COVID-19 pandemic. Therefore, the primary aim of this study was to investigate the differences in full and partial affect recognition between PBTSs and TD using two different measures of affect recognition (full facial features available and partial). The secondary aim was to examine the associations between affect recognition and social adjustment. We hypothesized that PBTSs would obtain lower scores on affect recognition with both full and partial facial cues compared to TD and that lower affect recognition would be associated with poorer social adjustment. The final exploratory aim was to investigate the association between affect recognition (full and partial) and exposure to masks during social interactions.

## 2. Materials and Methods

### 2.1. Participants

A convenient sample of 23 PBTSs and 24 TD participated, with one of their parents, in our cross-sectional study. The PBTSs had a mean age of 12.92 (SD = 2.46), a biological sex distribution of 60.9% male, and 91.3% were White. Their age at diagnosis ranged from 1 to 10 years old (M = 5.36, SD = 2.70), and their time since brain tumour diagnosis ranged from 1 to 14 years (M = 7.14, SD = 3.12). Diagnoses principally included medulloblastoma (34.8%), pilocytic astrocytoma (21.7%), and craniopharyngioma (17.4%). Primary medical treatment approaches included a combination of chemotherapy, radiation, and surgery (30.4%), surgery only (26.1%), and a combination of radiation and surgery (17.4%). The participating parents of the PBTSs were 87.0% female sex and 13.0% male sex, with a mean age of 42.72 (SD = 6.61) years old. The TD had a mean age of 11.50 (SD = 2.36) and a sex distribution of 50% female. The TD predominantly reported their race as White (50%), Black (12.5%), and Middle Eastern (20.8%). For the TD, their participating parents had a biological sex distribution of 91.7% female and 8.3% male, with a mean age of 41.83 (SD = 2.43) years old. Additional sample characteristics are summarized in Table 1.

Inclusion criteria for the PBTSs included being 8 to 16 years old, at least 1-year post-treatment, and French-speaking. French is an inclusion criterion as it is the official language of Quebec, Canada (where the study took place), and 77.5% of the population predominately speaks French at home [19,20]. Exclusion criteria included actively receiving treatment for relapse or palliative care, having an existing diagnosis of ASD, having first-degree relatives with ASD (i.e., higher likelihood of ASD traits), and a diagnosis of Tuberous Sclerosis Complex or Neurofibromatosis type 1 as there is an increased likelihood of also having ASD [21,22]. The exclusion criteria were selected given that significant affect recognition difficulties are well established in the ASD population, e.g., [23]. Due to the low base rate of pediatric brain tumours, children with any brain tumour diagnosis and all types of received treatments were eligible to participate. For TD, the inclusion criteria included being aged 8 to 16 years old and French-speaking. The exclusion criteria included having a medical history of any type of cancer, having an existing diagnosis of ASD, or having first-degree relatives with ASD (see reasons above).

### 2.2. Procedure

After receiving approval from the local institutional Research Ethics Board (#2022-3435), the recruitment of PBTSs and TD simultaneously took place from May 2022 to May 2023. During this time, mandatory masking was eliminated as of late June 2022 except in healthcare settings [24]. However, masks were still strongly recommended, with the Quebec Health Minister issuing a statement for this recommendation in November 2022 [25]. PBTSs were recruited either through provincial and national pediatric cancer organizations’ (Leucan and Brain Tumour Foundation of Canada) email listservs or through a local pediatric neuro-oncology clinical patient database. There were 72 eligible families in the database, and all were contacted either by phone or met in-person at the neuro-oncology clinic to discuss the study. From those, 30 families consented to participate, and 23 completed the study. Twenty families could not be reached (e.g., never responded to contact or no response to follow-up contacts). Twenty-two families declined to participate in the overall larger longitudinal study in which this study is included. Reasons for declining to participate included not having time (40.9%), not being interested in participating in research studies at this time (31.8%), and children not being interested in the research subject (27.3%). TD were recruited through advertisements shared online via the hospital’s website and social media platforms, email lists (e.g., staff listservs), Facebook groups (e.g., French parent groups), and community organizations (e.g., sports centres, libraries). Interested families were invited to contact a research assistant for more information. The families of 39 children responded to study advertisements, and 24 TD completed the study. Reasons families did not complete the study included the following: not being eligible after answering screening questions (20%), the research team receiving no responses to contact follow-ups about completing the study (46.7%), no longer being interested in participating (20%), or no longer having the time (13.3%).

After receiving detailed information about the study, interested and eligible families were asked to provide written assent/consent for their participation. Due to pandemic constraints and for accessibility purposes, all study measures were administered remotely. Children were scheduled for and participated in a one-on-one virtual study appointment (via Zoom) with a research assistant trained in the administration of all measures. In preparation for their appointment, families were asked to verify their internet access and received additional written instructions to optimize virtual assessment. For example, adhering to the Developmental Neuropsychological Assessment—Second Edition (NEPSY-II) telepractice guidelines by Pearson [26], participants were instructed to use a computer screen or a tablet for viewing images (i.e., not phones as the screen is too small), have good quality video and audio devices, and ensure their assessment environment are free of visual and physical distractions (e.g., no phones, pets, music). These were verified by the research assistant, with the help of parents, on the day of the appointment before presenting the study measures. During this meeting, children completed two performance-based measures (Affect Recognition subtest the NEPSY-II and the Reading the Mind in the Eye Test—Child Version [RMET]) and one self-reported questionnaire (COVID-19 Questionnaire on Social Interactions and Mask Exposure). For the NEPSY-II Affect Recognition subtest, the stimuli booklet was presented flat with a high-quality camera. In the case of the RMET, images were shared through screen-sharing. Parents’ participation consisted of individually completing a parent-reported questionnaire (see parent measures below) through the secure online platform LimeSurvey. 

### 2.3. Child Measures

#### 2.3.1. A Developmental Neuropsychological Assessment—Second Edition (NEPSY-II)

The NEPSY-II is a widely used, standardized measure of neuropsychological functioning [27,28]. For the current study, only the subtest of Affect Recognition was administered, which has acceptable validity and reliability (*r* ≥ 80 for children aged 7 to 16). This subtest consists of four different performance-based tasks assessing children’s ability to recognize affect (happy, sad, anger, fear, disgust, and neutral) from pictures of children’s full faces by matching a face displaying an emotion to another face displaying the same emotion [28]. A study indicated no significant score differences for young children (4–5 years old) between those who completed it in-person versus virtually, supporting that this measure may retain its original value when administered online [29]. Also, this subtest has been used in two recent studies with children with brain tumours newly diagnosed patients and preschoolers patients [30,31], suggesting it could be administered to children and adolescent PBTSs. Raw scores obtained were converted into aged-based standard scores (M = 10, SD = 3), with higher scores reflecting better ability to recognize affect. Norms for this subtest (2005–2006) were validated in 2012 with a sample of 185 French-speaking children [32].

#### 2.3.2. Reading the Mind in the Eye Test (RMET)

The RMET is a well-established performance-based measure, with adequate test–retest reliability [33] that we used as a proximal measure for affect recognition with partial facial expression availability. It was recently translated into Canadian French [34]. In the RMET, children are instructed to look at black-and-white static pictures of the eye region of various adult faces and select a word out of four proposed that best matches the emotion portrayed in the picture. It consists of 28 images, and the final raw score is based on the number of items the participant answered correctly [15]. Standardized scores for the child RMET are not currently available, but data from three studies with TD children of ages 6 to 17, published in 2014, 2015, and 2020 [35,36,37], indicate a weighted average of 17.78 (SD = 1.07).

#### 2.3.3. COVID-19 Questionnaire on Social Interactions and Mask Exposure

This short self-reported survey was created for this study to assess the frequency with which participants interacted with both adults and children wearing masks in the past month. As an example, questions for peers were as follows: “How many days a week do you interact (play, talk, do an activity), in person, with peers and friends?” and “When you interact with peers and friends, in person, how often do they wear masks?”. The same questions were asked for interactions with adults. Answer choices range from None/Never to Everyday/Always, depending on the question. The raw composite continuous score of total exposure to masks in social interaction reflects the number of days per week the child interacted with others (adults and peers) in the last month and the frequency with which others (adults and peers) wore masks during these interactions. This measure is included as Supplementary Materials.

### 2.4. Parent Measures

#### 2.4.1. Child Behaviour Checklist (CBCL)

The CBCL is a standardized parent-completed questionnaire consisting of various scales commonly used to detect emotional and behavioural problems in children and adolescents aged from 4 to 18 years old [38]. The CBCL has adequate validity and reliability, and it has been used in multiple studies with PBTSs, e.g., [39]. The Social Problems and Withdrawn subscales, which reflect difficulties in social adjustment [2,3], were used in this study. Higher standardized scores indicate more social difficulties as determined by the parent.

#### 2.4.2. Socio-Demographic Form and Medical Information

Parents completed a demographic questionnaire. Medical charts of PBTSs were also reviewed. Information on age, biological sex, race, time since diagnosis, and type of treatment (e.g., radiation, chemotherapy, surgery) of children was obtained. Information on parents’ marital status, educational background, and main occupation was also obtained to describe the sample.

### 2.5. Data Analysis

Descriptive statistics (e.g., n, sample percentage, means, standard deviation, range) summarizing group characteristics were generated for demographic information and medical information, as well as key study variables. As some studies propose using parametric tests with small sample sizes, e.g., [40,41], Pearson correlations were used to evaluate associations between continuous measures. The independent samples T-test (continuous data) and Chi-square test of homogeneity (proportions) were also performed to examine potential group differences between PBTSs and TD. To examine potential differences between full and partial affect recognition in the combined sample, a paired-sample T-test was conducted. To allow for comparison between the two measures, z-scores were generated for each affect recognition measure based on pre-pandemic norms and normative samples. Two one-sample T-tests were conducted to examine whether the current sample differed on affect recognition compared to pre-pandemic data. Cohen’s d was used to describe effect sizes. All analyses were conducted on SPSS, version 29.

## 3. Results

### 3.1. Preliminary Analyses (See Table 1 for Sample Characteristics)

#### Examination of Potential Group Differences and PBTS Associations with Medical Variables

The PBTSs and TD did not significantly differ in terms of age (*t*(45)= −2.01, *p* = 0.05, *d*= −0.59, 95% CI [−2.83, 0]) or sex (χ2(1) = 5.6, *p* = 0.45). Within the PBTSs, there was no significant difference in radiation (yes/no) on the NEPSY-II (*t*(21)= −0.18, *p* = 0.86, *d*= −0.08, 95% CI [−3.67, 3.09]) or the RMET (*t*(21) = 1.41, *p* = 0.17, *d* = 0.62, 95% CI [−1.16, 6.10]). Within the PBTSs, the time since diagnosis was not significantly associated with either full or partial affect recognition (NEPSY-II: *r* = −0.17, *p*= −0.17; RMET: *r* = 0.08, *p* = 0.08).

### 3.2. Primary (Main) Analyses

#### 3.2.1. Aim 1: Comparison of PBTSs and TD on Full and Partial Facial Affect Recognition

The results are shown in Table 2. Contrary to the hypothesis, there were no significant group differences between the PBTSs and TD in terms of either full or partial facial affect recognition. Therefore, the combined sample was used in subsequent analyses. 

#### 3.2.2. Aims 2 and 3: Associations of Affect Recognition with Social Adjustment and Mask Exposure

The results are shown in Table 3. No significant correlations were found between affect recognition (full and partial) with social adjustment, total mask exposure, or mask exposure by target (adult and peers wearing masks).

### 3.3. Secondary (Incidental) Findings

#### 3.3.1. Full versus Partial Affect Recognition

After generating z-scores for full and partial affect recognition based on normative data and reference means, a paired sample T-test indicated that there was a significant difference in the overall sample between scores on the NEPSY-II subtest and the RMET (*t*(46) = 6.46, *p* < 0.001, *d* = 0.94, 95% CI [1.95, 3.72]). Participants performed better on the partial affect recognition RMET task (M = 2.25, SD = 3.24) relative to the full facial affect recognition NEPSY-II task (M= −0.58, SD = 1.17).

#### 3.3.2. Pre-Pandemic Reference Norms versus Study Pandemic Sample

Our current pandemic sample obtained significantly lower scores on the NEPSY-II (M = 8.26, SD = 3.51) relative to pre-pandemic normative data (M = 10, SD = 3), *t*(46) = −3.41, *p* = 0.001, *d*= −0.50, 95% CI [−2.78, −0.71]). Our pandemic sample obtained higher scores on the RMET (M = 20.19, SD = 3.47) relative to a large comparison sample of children obtained before the pandemic weighted (M = 17.78, SD = 1.7 [35,36,37], *t*(46) = 4.77, *p* < 0.001, *d* = 0.70, 95% CI [1.39, 3.43]).

## 4. Discussion

The current study examined potential differences between PBTSs and TD on affect recognition while also exploring this skill’s associations with social adjustment and mask exposure. The results indicate that both groups have similar performances of affect recognition. In addition, it was found that affect recognition is not associated with either the sample’s social adjustment (social problems or withdrawn behaviours) or with their self-reported exposure to masks in their social interactions. Although they were not the main aims of the study, incidental findings show that the current sample demonstrated a better performance of partial affect recognition compared to full affect recognition. Similarly, the sample had better partial affect recognition compared to pre-pandemic data, yet worse full affect recognition.

Indeed, contrary to our hypothesis, PBTSs performed similarly on measures of full and partial facial affect recognition relative to TD. With the pandemic health recommendations (e.g., social distancing, lockdowns, mask-wearing, reduced number of people that one can interact with), both PBTSs and TD experienced, for a few years, a new social context with fewer interactions and similar opportunities to develop their social skills. Therefore, it is possible that the pandemic equalized opportunities between groups to practise their affect recognition. This finding is also consistent with two recent studies that found no differences in full affect recognition between PBTSs and TD with adult faces, [11] and with children with non-central nervous system solid tumours with child faces [14]. However, although our results are non-significant, effect sizes were small to medium in the direction of controls performing better, suggesting a trend toward potential better performance by the TD. These effect sizes indicate some alignment with studies that found significant differences with large effect sizes between PBTS and control groups [10,12], highlighting the need for further evaluation of these skills with larger samples. Notably, there was also more variability in the PBTS scores, suggesting a need for future exploration of risk and resilience factors in this population.

Inconsistent with previous PBTS research [10,11,12], the participants’ affect recognition was not associated with their social adjustment (social problems or withdrawn). Possibly, the pandemic may have affected parents’ responses on the subscales of social adjustment, which may have undermined possible correlations with that measure. Indeed, parents may have interpreted questions differently given altered patterns of socialization and external social concerns (e.g., activity restrictions and not the child’s behaviours). Another hypothesis for this result is that with the altered social context of the pandemic, children had to adjust the way they socialize, communicate, and the way they create and maintain relationships, e.g., [42,43]. Therefore, they may have needed to rely more heavily on other specific social skills such as better coping skills, social problem-solving, imagination to create social settings, and better online communication skills (e.g., social media use, instant messaging). Studies have found that online friend communication is a protective factor against loneliness and stress in adolescents [44] and that it is positively associated with the quality of existing friendships [45]. Hence, online communication skills may be currently more necessary for social adjustment than affect recognition. Additionally, we wonder whether a combination of social information processing skills may better influence social adjustment. Future research should thus consider examining many aspects of Yeates et al.’s [7] social competence model concurrently. 

Full and partial affect recognition were also found to not be correlated with total mask exposure in social interactions. A recent study similarly found that exposure to people wearing masks was not related to affect recognition from upper facial cues [46]. However, the amount of mask exposure in the past month, as assessed by our measure, may not be ideal for measuring this association. Rather, the long-term exposure to masks during the *years* of the pandemic might better show an association with affect recognition. Further, our data were collected in 2022–2023 when many mask mandates were no longer in effect, potentially limiting the weight of one-month mask exposure. Notably, both PBTSs and TD predominantly reported rarely or never interacting with others who wore masks at the time the study took place. 

Although we did not find associations of affect recognition with mask exposure, we hypothesize that practise effects from the *last few years* may still play a role. Indeed, incidentally, within our sample, participants appeared to perform better at recognizing affect with partial facial cues compared to full facial cues, possibly due to practise effects. At the outset of the pandemic, others have found that children were better at recognizing emotions from whole faces compared to masked faces, e.g., [47]. Our study was conducted toward the conclusion of the pandemic, following at least two years of various public health measures often mandating or encouraging mask use and social isolation, and therefore, there may be a higher likelihood of practise effects due to more time having passed with similar visual stimuli. 

Additionally, the combined current sample appeared to perform differently compared to pre-pandemic normative data and reference samples. Our current sample seemed to have a more difficult recognizing affect with full facial features compared to normative data. The pandemic context (e.g., limited social interactions, mask-wearing) might have negatively influenced the development of full-face affect recognition in youth during a period of typical improvement in learning this specific skill, e.g., [48]. Conversely, our sample seemed to have better partial affect recognition relative to reference samples pre-pandemic, which is consistent with a study that found that adolescents tested during the COVID-19 pandemic obtained higher scores on the adult RMET compared to a sample tested before the pandemic [46]. This finding may be explained by practise effects. Indeed, the development of affect recognition is influenced by stimuli presented to children, e.g., [49]. With the pandemic, the current sample interacted with others wearing masks more frequently than pre-pandemic samples. They thus had more opportunities to practise recognizing emotions solely based on eye-region facial cues. While practise effects are one hypothesis for this incidental finding, it is essential to consider potential limitations arising from comparing different samples at different time points. Disparities in samples and possible enrollment bias in our sample restrict the conclusion of true pre- and post-pandemic differences in affect recognition. Also, this result could be explained by the difference between virtual and in-person administration between pre-pandemic data and our sample, although a study found that children aged 4–5 performed similarly on the NEPSY-II Affect Recognition subtest online compared to face-to-face [29].

The present study offers several strengths. First, this study has a comparison group composed of TD, allowing us to evaluate PBTS skills relative to their peers. Second, this study innovatively examined the potential influence of the COVID-19 pandemic on affect recognition, by both using a measure of eye-region affect recognition (similar to facial stimuli with masks) and considering mask exposure as a variable. Third, all measures were remotely administered. Virtual assessments may have allowed the recruitment of more rural PBTSs, easier accessibility, and more flexibility for families. Fourth, the use of the NEPSY-II subtest offers many advantages such as its diverse visual stimuli, standardized scores, and easy clinical use, as it is embedded within an established and widely used neuropsychological assessment battery [28].

The limitations of our study also need to be considered. First and most importantly, our small sample size limits the statistical power of the executed analyses and the conclusions to be made about PBTS experiences. Although our institution is the largest pediatric brain tumour centre in Quebec, obtaining large PBTS samples is often challenging due to the low base rate of brain tumours, mortality rate, and the typically single-site nature of novel exploratory studies. Future studies with larger sample sizes are necessary, and we are hopeful regarding the efforts of researchers to collaborate and partake in multi-site studies to address this issue, e.g., [50]. Second, there is a potential sample bias, especially for TD. It is possible that parents of TD with more social concerns were more motivated to participate in this project. Third, although measures were carefully chosen to answer study questions, several weaknesses in our measurement approach may still be noted. For instance, the RMET is only a proxy for affect recognition with partial facial cues and does not have standardized scores. Both our affect recognition measures are also considerably different (e.g., different tasks, one with child faces and the other adult faces, colour versus black-and-white stimuli), limiting the true comparison between full and partial affect recognition. The CBCL, although widely used and validated, is a parent-reported questionnaire and has limitations such as its low agreement to peer reports [51]. The COVID-19 Questionnaire on Social Interactions and Mask Exposure is an exploratory measure created for this study and did not capture the overall history of mask exposure or the total number of contacts per day with people wearing masks, reducing the representativeness of the influence of mask exposure. Other validated questionnaires on the impact of the pandemic should be considered for future research, e.g., [52]. Lastly, this study used virtual assessments, which offer advantages but also certain limitations such as less control of the assessment environment. Although it is possible that results were influenced by the virtual nature of the assessment, others have found that youth perform similarly on the NEPSY-II Affect Recognition subtest online compared to face-to-face [29].

## 5. Conclusions

Considering the importance of social competence, e.g., [3,53], and the well-established social difficulties experienced by PBTSs [2,3], it is essential to better understand the potential underlying processes of these impairments in this clinically vulnerable population. In addition, the global pandemic has added a pressing need to attend to its influence on the social development of children and adolescents. Therefore, the current study represents a step toward a better understanding of PBTS facial affect recognition, within the context of the pandemic. It informs an array of future directions for investigation. First, longitudinally determining the impact of the pandemic on the social development of TD children and PBTS is necessary. As hypothesized, certain social skills may now be more important as youth needed to adapt to their changing social environment. Creating new norms for measuring social competence post-pandemic may be needed. For instance, social competence measures could incorporate the various ways in which youth have increasingly learned to interact virtually or via social media platforms. The role of digital literacy on social adjustment appears largely unexplored in PBTSs to date. 

## Figures and Tables

**Table 1:** Sample demographic information.

	PBTS (n = 23)	TD (n = 24)
	n (%)	
**Race**		
White	21 (91.3)	13 (54.2)
Latino/Hispanic	1 (4.3)	
Black		2 (8.3)
Middle Eastern		4 (16.7)
North African	1 (4.3)	3 (12.5)
Multiracial		2 (8.3)
**Parent marital status**		
Common-law	7 (30.4)	9 (37.5)
Married	10 (43.5)	15 (62.5)
Divorced or separated	5 (21.7)	
Widowed	1 (4.3)	
**Parent completed education**		
High School Diploma	5 (21.7)	
Diploma of Vocational Studies	1 (4.3)	
College diploma	4 (17.4)	3 (12.5)
Bachelor’s degree	11 (47.8)	10 (41.7)
Master’s degree	2 (8.7)	11 (45.8)
**Parent main occupation**		
Full-time work	18 (78.3)	16 (66.7)
Part-time work	3 (13.0)	2 (8.3)
Unemployed/Looking for work	1 (4.3)	2 (8.3)
Student	1 (4.3)	3 (12.5)
Self-employed		1 (4.2)
**Tumour type**		
Medulloblastoma	8 (34.8)	
Ependymoma	4 (17.4)	
Craniopharyngioma	4 (17.4)	
Pilocytic astrocytoma	5 (21.7)	
Other	2 (8.7)	
**Tumour Treatment**		
Chemotherapy only	2 (8.7)	
Radiation only	1 (4.3)	
Surgery only	6 (26.1)	
Chemotherapy and radiation	1 (4.3)	
Chemotherapy and surgery	2 (8.7)	
Radiotherapy and surgery	4 (17.4)	
Chemotherapy, radiation, and surgery	7 (30.4)	

**Table 2:** Differences between PBTSs and TD on affect recognition.

	PBTS (n = 23)		TD (n = 24)		Independent Samples T-Test
	M ± SD	Range	M ± SD	Range	
NEPSY-II, Affect Recognition Subtest	7.57 ± 3.63	1–13	8.92 ± 3.34	2–16	*t*(45) = 1.33, *p* = 0.19, *d* = 0.39, 95% CI [−0.69, 3.40]
RMET	19.39 ± 4.08	10–24	20.96 ± 2.63.	17–27	*t*(37.36) = 1.56, *p* = 0.13, *d* = 0.46, 95% CI [−0.47, 3.60]
CBCL, Social Problems Subscale	58.87 ± 7.64	50–72	54.33 ± 5.43	50–66	
CBCL, Withdrawn Subscale	58.52 ± 9.92	50–85	55.38 ± 7.47	50–78	

Note. The PBTS NEPSY-II Affect Recognition mean was within the below-average range, while the mean of the TD was within the average range [28]. The means scores of PBTSs and TD were within the normal range of the CBCL Social Problems and Withdrawn subscales (t-score of 65 and below) [38], although the TD were approximately half and the PBTSs, a full standard deviation above the normative mean.

**Table 3:** Associations of affect recognition with social adjustment and mask exposure in the combined sample.

	CBCL, Social Problems	CBCL, Withdrawn	Total Mask Exposure	Adult Mask Exposure	Peer Mask Exposure
NEPSY-II, Affect Recognition Subtest	*r* = −0.12, *p* = 0.43	*r* = −0.03, *p* = 0.83	*r* = 0.22, *p* = 0.14	*r* = 0.23, *p* = 0.12	*r* = 0.16, *p* = 0.29
RMET	*r*= −0.21, *p* = 0.15	*r*= −0.23, *p* = 0.12	*r*= 0.17, *p* = 0.27	*r* = 0.25, *p* = 0.09	*r* = 0.04, *p* = 0.77

## Data Availability

Data presented in this article are not readily available because that data are part of an ongoing study, and participants did not consent for their information to be publicly shared.

## Supplementary Materials

The following supporting information can be downloaded at: https://www.mdpi.com/article/10.3390/curroncol31080339/s1, COVID-19 Questionnaire on Social Interactions and Mask Exposure.
