# Supplementary material for: Full and Partial Facial Affect Recognition in Pediatric Brain Tumour Survivors and Typically Developing Children Following COVID-19 Pandemic
Source: Curr Oncol. 2024 Aug 9;31(8):4546–58. doi: 10.3390/curroncol31080339 (PMC11353234; doi:10.3390/curroncol31080339)
Supplement: Supplementary file 1 [file curroncol-31-00339-s001.zip › curroncol-3095053-supplementary.pdf]

COVID-19 Questionnaire sur les interactions sociales et les masques

Voici un court questionnaire sur tes interactions sociales du dernier mois et les masques durant la COVID-19. Il n'y a pas de bonnes ou de mauvaises réponses. Nous voulons seulement en apprendre plus sur tes expériences. S'il te plaît, réponds aux questions le plus honnêtement possible. Comme rappel, toutes tes réponses sont confidentielles.

Interactions sociales, **en personne**, avec des adultes (autres que tes parents)

Choisis et encercle la réponse selon tes expériences **du dernier mois**.

1. Combien de jours par semaine interagis-tu (par exemple, jouer ensemble, parler ensemble, faire une activité ensemble), **en personne**, avec des adultes (autres que tes parents)?

Jamais	Un ou deux jours par semaine	Trois ou quatre jours par semaine	Cinq ou six jours par semaine	À tous les jours

2. Lorsque tu interagis avec des adultes (autres que tes parents) **en personne**, à quelle fréquence portent-ils des masques?

Jamais	Rarement	Parfois	Souvent	Toujours

3. Est-ce que ton enseignant(e) porte un masque pour enseigner **en personne** (pas en ligne)?

Oui	Non

4. À quel niveau les masques te dérangent-ils lors de tes interactions sociales avec des adultes (autres que tes parents)?

Pas du tout	Pas beaucoup	Un peu	Beaucoup	Ne s'applique pas (les gens avec qui j'interagis ne portent pas de masques)

Interactions sociales, **en personne**, avec des camarades de classes et/ou des ami(e)s)

Choisis et encercle la réponse selon tes expériences du **dernier mois**.

5. Combien de jours par semaine interagis-tu (par exemple, jouer ensemble, parler ensemble, faire une activité ensemble), **en personne**, avec des camarades de classes et/ou des ami(e)s)?

Jamais	Un ou deux jours par semaine	Trois ou quatre jours par semaine	Cinq ou six jours par semaine	À tous les jours

6. Lorsque tu interagis avec des camarades de classes et/ou des ami(e)s **en personne**, à quelle fréquence portent-ils des masques?

Jamais	Rarement	Parfois	Souvent	Toujours

7. Est-ce que vous devez porter un masque en classe, **en personne** (pas en ligne)?

Oui	Non

8. À quel niveau les masques te dérangent-ils lors de tes interactions sociales avec des camarades de classes et/ou des ami(e)s?

Pas du tout	Pas beaucoup	Un peu	Beaucoup	Ne s'applique pas (les gens avec qui j'interagis ne portent pas de masques)

<u>Bien-être durant la COVID-19</u>

En général, comment la COVID-19 a-t-elle affecté chacune des situations suivantes?

9. Ton bien-être social—les relations avec les adultes dans ta vie (membres de ta famille, enseignant(e)s, ou autres).

Rendu beaucoup mieux	Rendu un peu mieux	Rendu un peu pire	Rendu vraiment pire	N'est pas applicable

10. Ton bien-être social—les relations avec tes camarades de classes et/ou tes ami(e)s.

Rendu beaucoup mieux	Rendu un peu mieux	Rendu un peu pire	Rendu vraiment pire	N'est pas applicable

11. Ton bien-être émotionnel—anxiété/inquiétude

Rendu beaucoup mieux	Rendu un peu mieux	Rendu un peu pire	Rendu vraiment pire	N'est pas applicable
